# Association of extensively drug resistant salmonella infection in children with typhoid fever

**DOI:** 10.12669/pjms.38.7.5868

**Published:** 2022

**Authors:** Hanif Memon, Farhan Saeed, Muhammad Iqbal, Erum Saboohi, Shahina Hanif, Abdul Hadi Hassan Mallick

**Affiliations:** 1 Prof. Dr. Hanif Memon, MBBS, DCH, FCPS. Professor of Paediatrics, Karachi Adventist Hospital, Karachi, Pakistan; 2 Dr. Farhan Saeed, MBBS, DCH, FCPS. Associate Professor of Paediatrics, Liaquat College of Medicine and Dentistry, Karachi, Pakistan; 3 Dr. Muhammad Iqbal, MBBS, MRCPCH. Senior Registrar Pediatrics, Liaquat College of Medicine and Dentistry, Karachi, Pakistan; 4 Dr. Erum Saboohi, MBBS, MD. Assistant Professor of Pediatrics, Sir Syed College of Medical Sciences for Girls & Sir Syed Hospital, Karachi, Pakistan; 5 Dr. Shahina Hanif, MBBS, DCH, MCPS, FCPS. Professor of Paediatrics, United Medical & Dental College Karachi, Karachi, Pakistan; 6 Abdul Hadi Hassan Mallick Fourth year MBBS Student, Dow Medical College, Dow University of Health Sciences, Karachi, Pakistan

**Keywords:** XDR enteric fever, Salmonella infection, Pediatric

## Abstract

**Objectives:**

In resource limited countries facing a huge burden of multidrug resistant and extensively drug resistant (XDR) enteric fever, treatment is a great challenge on the part of a patient as well as a health care professional. This study was conducted to determine the association of XDR enteric fever with various studied factors among hospitalized culture-positive pediatric patients in a tertiary care hospital setup.

**Methods:**

We conducted a descriptive observational study at The Karachi Adventist Hospital from July 01, 2019, to March 31, 2020 on 143 hospitalized children with culture proven enteric fever who were already on empirical antibiotics. Depending on the variability of the course of illness and clinical responses to given antibiotics, the data was gathered on a structured data sheet. Association of various study parameters and their significance in relation to XDR salmonella infection was analyzed and studied.

**Results:**

The age group highly affected was 5-7.5 years, with a male preponderance of 61.5%. Majority were from urban slums areas of Karachi (53.8%) and 52% were admitted between 7 to 14 days of fever onset. XDR salmonella infection was observed in 79% of blood culture isolates. None of the XDR patients were consuming boiled water and neither of these infected children were vaccinated against salmonella typhi. Duration of fever before hospitalization, non-consumption of boiled or mineral water, ciprofloxacin use and lack of typhoid vaccination showed statistically strong association with XDR enteric fever (p<0.01).

**Conclusion:**

Prehospitalization fever duration, use of boiled/mineral water, ciprofloxacin use and typhoid vaccine status showed strong association with XDR salmonella infection. Prioritizing the focus on healthcare awareness, early access to proper health care facility, discouraging over-the-counter drugs and enforcement of immunization will help decline the dissemination of this dreadful disease.

## INTRODUCTION

Enteric fever is a term collectively used for typhoid and paratyphoid fevers. *Salmonella enterica* serovar Typhi (S. Typhi) is responsible for typhoid fever while paratyphoid fever is caused by *Salmonella enterica* serovars Paratyphi A, B, and C.^1^ The incidence of typhoid fever is 100-1,000 cases per 100,000 population in the developing world.^2^

Annually, typhoid fever affects 21.6 million people worldwide resulting in 216,500 deaths, majority of them from Asia. Increasing cases of antibiotic resistance as well as multiple drug resistance (MDR) have been reported in Indian Sub-continents.^3^ Another study mentioned Asia as an endemic region for typhoid fever, having a majority of cases from Pakistan, Bangladesh, India and China.^4^

Faeces is the main source of transmission for salmonella typhi organism.^5^ Typhoid patients often present with fever, headache, gastrointestinal symptoms, and occasionally with complications like gastrointestinal bleeding, perforation, peritonitis and meningo-encephalitis.^6^ Anemia, thrombocytopenia, leucopenia, eosinophilia and sub clinical disseminated intravascular coagulation are frequently seen in enteric fever.^7^ Due to excessive use of antibiotics, the salmonella pathogen isolation is reduced in blood and faeces.

Extensively Drug Resistant (XDR) typhoid fever includes typhoid caused by typhi strains that are resistant to all recommended antibiotics for typhoid fever but is sensitive to carbapenems and azithromycin. If the patient is clinically stable, azithromycin for 7-10 days is recommended. If the patient is hemodynamically unstable, Imipenem, Meropenem, Ertapenem for 10 – 14 days treatment is recommended.^8^ includes typhoid caused by typhi strains that are resistant to ampicillin, trimethoprim-sulfamethoxazole, chloramphenicol, and/or fluoroquinolones. Intravenous ceftriaxone can be switched to oral cefixime once the child is afebrile and able to tolerate oral medications. Therapy with cephalosporin is recommended for at least for 14 days.^8^ Certain in-vitro studies have shown tigecycline, cefpodoxime and cefpirome as potential therapeutic agents resistant strains of salmonella Typhi.^9^

The prevalence of antibiotic-resistant bacteria is an increasing problem for the treatment of typhoid fever, especially in the developing world including Pakistan. In 2017, a large outbreak emerged in Sindh, Pakistan for *S*. Typhi showing resistance to classical first- and second-line antibiotics but sensitive only to azithromycin, carbapenems and tigecycline.^10^ In another study, the author found that most of the affected individuals were children under 15 years.^11^ The main reason for the outbreak and extensively drug-resistant typhoid was the mixing of drinking water with sewage, decreased rate of typhoid vaccination and overpopulation.^12^ In March 2018, the WHO recommended the use of typhoid conjugate vaccine in children aged six months or older, in typhoid endemic regions or countries with antimicrobial resistance.^13^

The aim of this study was to determine the factors that are strongly associated with XDR typhoid in our study population.

## METHODS

This descriptive prospective observational study was carried out at pediatric department of The Karachi Adventist Hospital. Ethical Committee approval taken on May 30,2019 with IRB number, IRB-KAH-005/2019.The sample size was calculated using online sample size calculator for proportion at www.openepi.com^14^ (version 3.01). The estimated sample size was 143 with 5% margin of error and 95% confidence interval.

We used a non-probability purposive sampling plan. All patients (n=143) aged six months to 13 years who were admitted from July 01, 2019, to March 31, 2020; with complaints of fever and subsequently diagnosed with salmonella with a positive culture, were included in the study. Patients <6 months of age, with a negative culture for salmonella, and fever due to any other cause were excluded from the study. For the ease of description, we used terms XDR for ’Extensively Drug Resistant’ salmonella infection and non-XDR for MDR and non-MDR strains of salmonella typhi.

Data was collected once the patient was hospitalized, and when blood culture and sensitivity (C/S) report was available. Antibiotic usage before hospitalization (cefixime, co amoxiclav, ciprofloxacin) was documented. Patients who came without any prior treatment with an antibiotic, were prescribed Augmentin (amoxicillin/clavulanate) intravenously, considering them as a case of uncomplicated enteric fever,^8^ and those who took Augmentin prescribed at recommended dose were empirically given I/V ceftriaxone, considering them as a MDR case^8^ till the culture & sensitivity reports were available, and then changed the antibiotics accordingly and continued the antibiotics for four days more after the child become afebrile. Those patients who showed high drug resistance initially added with Intravenous Azithromycin to the existing treatment four days when child became afebrile, then further continued the same drug for three more days. In case of persistent fever even after four days Augmentin (amoxicillin/clavulanate) or ceftriaxone were discontinued but Azithromycin was continued for three more days. Injection meropenem was added and the treatment was continued with meropenem till the child became afebrile and then the treatment was further extended for four more days (after child became afebrile).

**Figure:**
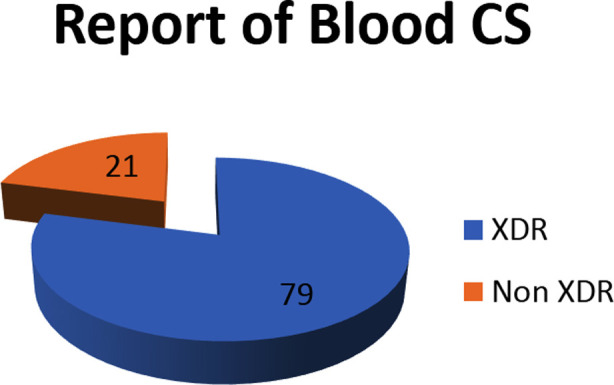
Fig.1

Data were stored and analyzed using IBM SPSS version 23.0. Counts with percentages were reported for baseline characteristics of studied samples. Pearson Chi Square test was used to check the association of blood CS with parent’s education, duration of fever at admission, type of water consumption, edibles from Dhabas or restaurants, symptoms associated with fever and status of typhoid vaccination. P-values less than 0.05 were considered statistically significant. Pie chart and bar diagrams were also used to give graphical presentation of data.

## RESULTS

Baseline characteristics of studied samples are reported in Table -1, which showed that among 143 children, 19.6% were belonging to age group 5 – 7.5 years old, 61.7% were male gender, 46.2% from urban areas, 34.3% parents had private services, 30.1% mothers found with up to secondary or higher secondary education level whereas 39.2% fathers found with up to secondary / higher secondary education.

**Table I:** Baseline Characteristics of Studied Samples (n=143).

Characteristics		N	%
Age of patient	0.5-2 year	15	10.5
	2-3.5 years	16	11.2
	3.5-5 years	25	17.5
	5-7.5 years	28	19.6
	7.5-9 years	18	12.6
	9-10.5 years	23	16.1
	10.5-12 years	18	12.6
Gender of patient	Male	88	61.5
	Female	55	38.5
Residence of patient	Urban	66	46.2
	Urban slums	77	53.8
Occupation of parent/guardian	Laborer	59	41.3
	Shop keeper	22	15.4
	Driver	10	7.0
	Private service	49	34.3
	Government service	3	2.1
Education of mother	Illiterate	35	24.5
	Primary	41	28.7
	Secondary/ higher secondary	43	30.1
	Graduate	24	16.8
Education of father	Illiterate	18	12.6
	Primary	28	19.6
	Secondary/ higher secondary	56	39.2
	Graduate	41	28.7

Association of blood CS with studied factors is shown in Table-II, where among XDR samples 24.8% mother found illiterate, 14.2% father found illiterate, 52.2% Children found with 7-14 days duration of fever at admission, 95.6% samples found multiple symptoms associated with fever, 38.9% took cefixime, 42.5% took Co amoxiclav and 39.2% took Ciprofloxacin as antibiotics, whereas among non XDR samples 23.3% mother, 10% father found illiterate 83.3% found with 7-14 days duration of fever at admission, all of the non XDR samples were found with multiple symptoms associated with fever. Pearson chi square test showed a significant association of XDR and non XDR samples with duration of fever at admission, and ciprofloxacin medicine with blood c/s outcomes (P<0.01).

**Table II:** Association of Blood C/S with Studied Factors.

Factors		Report of Blood C/S				

		XDR (n=113)		NON XDR (n=30)		p-value

		N	%	N	%	
Education of mother	Illiterate	28	24.8	7	23.3	0.31
	Primary	32	28.3	9	30.0	
	Secondary / Higher secondary	37	32.7	6	20.0	
	Graduate	16	14.2	8	26.7	
Education of father	Illiterate	15	13.30	3	10.0	0.42
	Primary	25	22.1	3	10.0	
	Secondary / Higher secondary	42	37.2	14	46.7	
	Graduate	31	27.4	10	33.3	
Duration of fever at admission	Less than 7 days	7	6.2	1	3.3	<0.01*
	7-14 days	59	52.2	25	83.3	
	More than 14 days	47	41.6	4	13.3	
Symptoms associated with fever	Anorexia	3	2.7	-	-	0.50
	Diarrhea	2	1.8	-	-	
	Multiple symptoms	108	95.6	30	100.0	
Cefixime	Yes	44	38.9	16	53.3	0.15
	No	69	61.1	14	46.7	
Coamoxiclav	Yes	48	42.5	17	56.7	0.16
	No	65	57.5	13	43.3	
Ciprofloxacin	Yes	45	39.8	19	63.3	0.02*
	No	68	60.2	11	36.7	

* p<0.05 was considered significant using Pearson Chi Square test.

**Fig.2:**
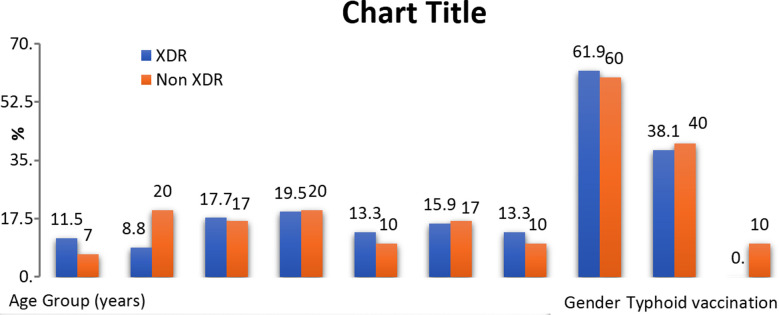
Showing among XDR samples 19.5% children were age group under 5 – 7.5 years old, 61.9% were male gender and none of XDR child vaccinated for typhoid.

Among the XDR samples, as shown in Table-III, 68.1% subjects used boiled water, 56.6% uses mineral water, 48.7% uses R/O plant water, 65.5% take food from Dhabas, 43.3% take food from restaurants and none of the sample was vaccinated for typhoid. There was a significant association observed for use of boil water, mineral water and patients’ status of typhoid vaccination with p<0.05.

**Table III:** Association of Blood CS with Studied Risk Factors.

Risk Factors		Report of blood CS				

		XDR (n=113)		Non-XDR (n=30)		p-value

		N	%	n	%	
Use of boiled water	Yes	77	68.1	12	40	<0.01*
	No	36	31.9	18	60	
Use of mineral water	Yes	64	56.6	9	30	<0.01*
	No	49	43.4	21	70	
Use of R/O plant water	Yes	55	48.7	11	36.7	0.24
	No	58	51.3	19	63.3	
Food from dhabas	Yes	74	65.5	21	70	0.64
	No	39	34.5	9	30	
Food from restaurants	Yes	49	43.4	19	63.3	0.052
	No	64	56.6	11	36.7	
Patient status of typhoid vaccination	Yes	0	0.0	3	10.0	<0.01*
	No	113	100	27	90.0	

* p<0.05 was considered significant using Pearson Chi Square test.

## DISCUSSION

Typhoid fever is an ever-feared illness in our part of the world with the increasing cases of emerging multidrug resistance and quinolone resistance in the last few years.^15^ In the current study, statistically strong association with XDR salmonella was seen with duration of fever before admission, consumption of water without boiling or filtering and status of typhoid vaccination.

In our study the age range of the children most affected is 5-9 years (32.2%), a vulnerable age group because of easy access to street vendors and lack of awareness regarding hygiene in these immature minds. This finding is consistent with the study done by Saeed N et al. in Pakistan in 2019 where 33% of the enrolled subjects were between ages five to 10 years.^16^ Similar findings were observed in a study done by Iftikhar A and Colleagues in a tertiary care hospital in Lahore in 2018 where 52% of the affected patients aged 5- 10 years.^17^

There was a clear male predominance in the enrolled subjects in our study (61.5%) because of more outdoor exposure, carefree nature, and risk-oriented attitude in boys. The male predominance is consistent with the findings in a previous study by Khan and Mohammad in 2012^18^. Among the enrolled subjects 53.8% were from urban slums. Belonging to urban slums, low socioeconomic status, considering symptoms as mild, using herbs as home remedies are some of the reasons for delay in approaching appropriate healthcare facilities.^19^

Unsafe drinking water has been an identifiable risk factor for enteric fever causation since long.^20^ Our study showed significant association of non-consumption of boiled and standard mineral water in XDR causation. Similar finding was also studied by Vijayalaxmi V. Mogasale and colleagues in 2018 where unsafe and unimproved drinking water consumption had direct correlation with enteric fever.^21^ M. Imran Khan and colleague also observed significant relation of enteric fever to lack of access to safe water in Karachites.^22^

High grade fever is the most common clinical manifestation of enteric fever especially in pediatric age groups which aggravates parental anxiety.^23^ In our study 58.7% patients had fever for seven to 14 days before the admission. Majority of them had temperature of more than 100^o^F. This observation is comparable with the retrospective review done by Parry et al where the median duration of fever at admission of enteric fever cases was 7 days *(IQR*, 5–14 days).^24^

Our study showed highly significant association with vaccine status and XDR positivity suggesting effective role of TCV against disease causation. This observation is supported by the work done by Rabab Batool, et al. and Yousufzai, et al. in 2021 where they found TCV effectiveness among children and adolescents in XDR typhoid Outbreak.^25^,^26^

Enteric fever is an infection manifesting multi-system involvement and rapid disease progression. Same holds true for our enrolled subjects where majority (96.5%) presented with multiple symptoms i.e., lengthy fever (41.6%), abdominal pain, anorexia, vomiting, diarrhea, myalgias, headaches (95.6% collectively) etc. These observations are consistent with the research done by Dekisisa, T., Gebremedhin, E.Z. in Ethiopia and an analysis in BMJ.^1^,^20^

### Limitation

It includes single center, scarcity of literature on unhealthy eating, TCV vaccine and R/O water consumption. Further studies should be conducted in other towns of Karachi and Pakistan highlighting the association of population density, use of self-medication and over the counter drugs with XDR salmonella infection.

## CONCLUSION

The burden of XDR enteric fever is on the rise. Our study showed strong association of XDR salmonella infection with long duration of fever, use of unsafe drinking water and vaccination status of admitted children. Multi-drug resistance is the main concern in XDR enteric fever treatment which needs to be resolved by effective community level preventive measures like hygienic living, use of safe water for drinking, promotion of conjugate typhoid vaccine and health care level effective strategies.

### Authors’ Contribution

**MHM:** Concept, design, approval, critical review. **FS:** Concept, Design, approval. **MI:** Acquisition of data, drafting, critical review. **ES:** Drafting, critical review, accuracy and integrity. **SH:** Drafting, data analysis, critical review. **AHHM:** Drafting, data analysis, critical review.
